# Adhesional Omental Hernia With Small Bowel Obstruction in an Elderly Patient With a Virgin Abdomen: A Case Report

**DOI:** 10.7759/cureus.43365

**Published:** 2023-08-12

**Authors:** Saw Ping Wei, Noor Khairiah Abdul Karim, Mohd Ezane Aziz, Tan Shong Sheng

**Affiliations:** 1 Department of Radiology, Hospital Universiti Sains Malaysia, Kubang Kerian, MYS; 2 Department of Radiology, Advanced Medical and Dental Institute, Universiti Sains Malaysia, Bertam, MYS; 3 Department of Radiology, Universiti Sains Malaysia/Hospital Universiti Sains Malaysia, Kubang Kerian, MYS; 4 Department of Surgery, Hospital Universiti Sains Malaysia, Kubang Kerian, MYS

**Keywords:** congenital band, internal hernia, virgin abdomen, small bowel obstruction, adhesional omental hernia

## Abstract

Small bowel obstruction is a frequently encountered surgical condition in adults. Its most prevalent causes include adhesions resulting from prior abdominal surgery or peritoneal infection. However, cases of small bowel obstruction caused by omental bands in elderly individuals with no prior abdominal surgeries are exceedingly rare, with only a few reported in the literature. Here, we report a case of an elderly patient with small bowel obstruction caused by internal herniation through an omental band, without prior abdominal surgery or trauma. The initial impression was mesenteric ischemia, which posed a diagnostic dilemma as the patient did not exhibit any clinical risk factors for mesenteric ischemia with absent history of previous trauma or abdominal surgery. A computed tomography (CT) scan was performed, revealing clustering of small bowel loops in the right hemiabdomen with mild dilatation and a close loop configuration indicative of an internal hernia. Internal hernias are rare and challenging to diagnose clinically as they lack specific signs and symptoms. In this case, CT played a crucial role in enabling preoperative diagnosis of an internal hernia and guiding early management.

## Introduction

Small bowel obstruction in adults can result from various factors, including adhesion, hernias, and neoplasms. Among them, internal hernias account for 0.6 to 5.8% of all small bowel obstructions [1]. Adhesion is the most frequent cause, representing 65-75% of cases, with congenital omental bands contributing to 3% of cases [2,3]. The formation of omental bands, which can result from iatrogenic peritoneal injury or be congenital in pediatric patients, is a notable condition. While most omental bands are associated with previous surgeries. particularly bariatric procedures, there are rare cases where senile atrophy of the omentum can lead to this, even in individuals without a history of abdominal surgery [4].

## Case presentation

A 76-year-old Malay gentleman with no known medical illnesses presented with acute symptoms of vomiting and periumbilical pain radiating to the epigastric region for two days. The abdominal pain was cramping in nature, and the pain score was graded as 10. Notably, the patient had experienced constipation for the past three days but had no constitutional symptoms or family history of malignancy. Vital signs showed a slightly hypertensive state with a blood pressure of 145/86 mmHg and an irregularly irregular heart rhythm but not tachycardic. Physical examination revealed a distended abdomen with tenderness over the epigastric and right hypochondrium while hernial orifices remained intact and no mass was detected during digital rectal examination. 

**Figure 1:**
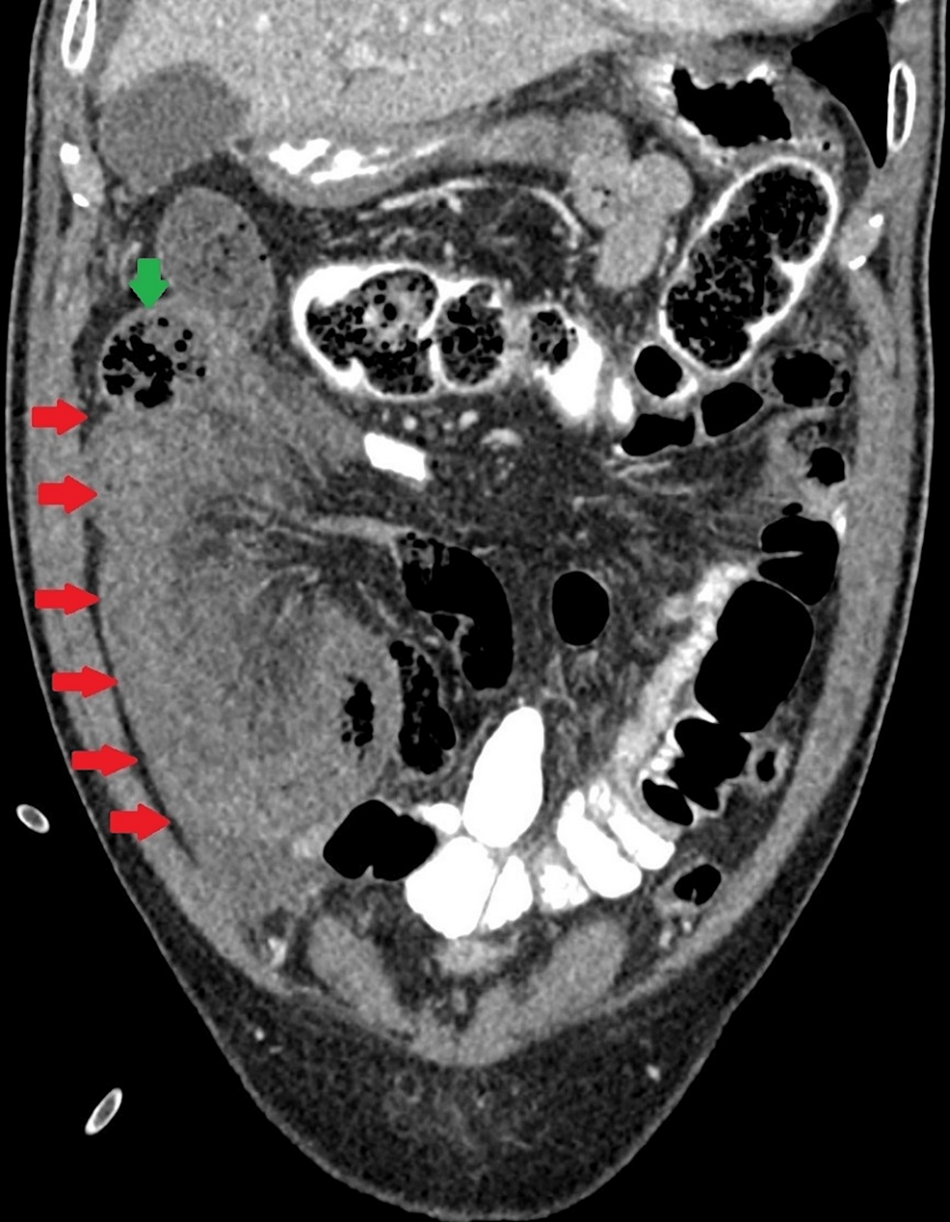
Coronal view of a computed tomography (CT) scan revealing C-shaped clustered loops of the ileum (red arrows) converging medially. Presence of small bowel feces sign (green arrow).

Laboratory findings indicated a white blood cell count of 11.0x 109/L, a hemoglobin level of 14.1 g/dl, and normal blood urea and electrolyte values. Amylase showed 103 U/L and venous blood lactate levels were elevated at 4.6 mmol/L. Electrocardiogram was performed and revealed atrial fibrillation. Chest X-ray revealed no air under the diaphragm. The presence of sentinel loops in the epigastric and paraumbilical regions on plain abdominal X-rays prompted further investigations. A subsequent computed tomography (CT) abdomen scan provided crucial insights, revealing clustered C-shaped loops of the distal ileum converging toward a mesenteric defect in the right paramedian region as shown in Figure 1. Additional findings such as the small bowel feces sign, thickened bowel wall of the contained ileal loops, and adjacent mesenteric streakiness were also observed.

**Figure 2:**
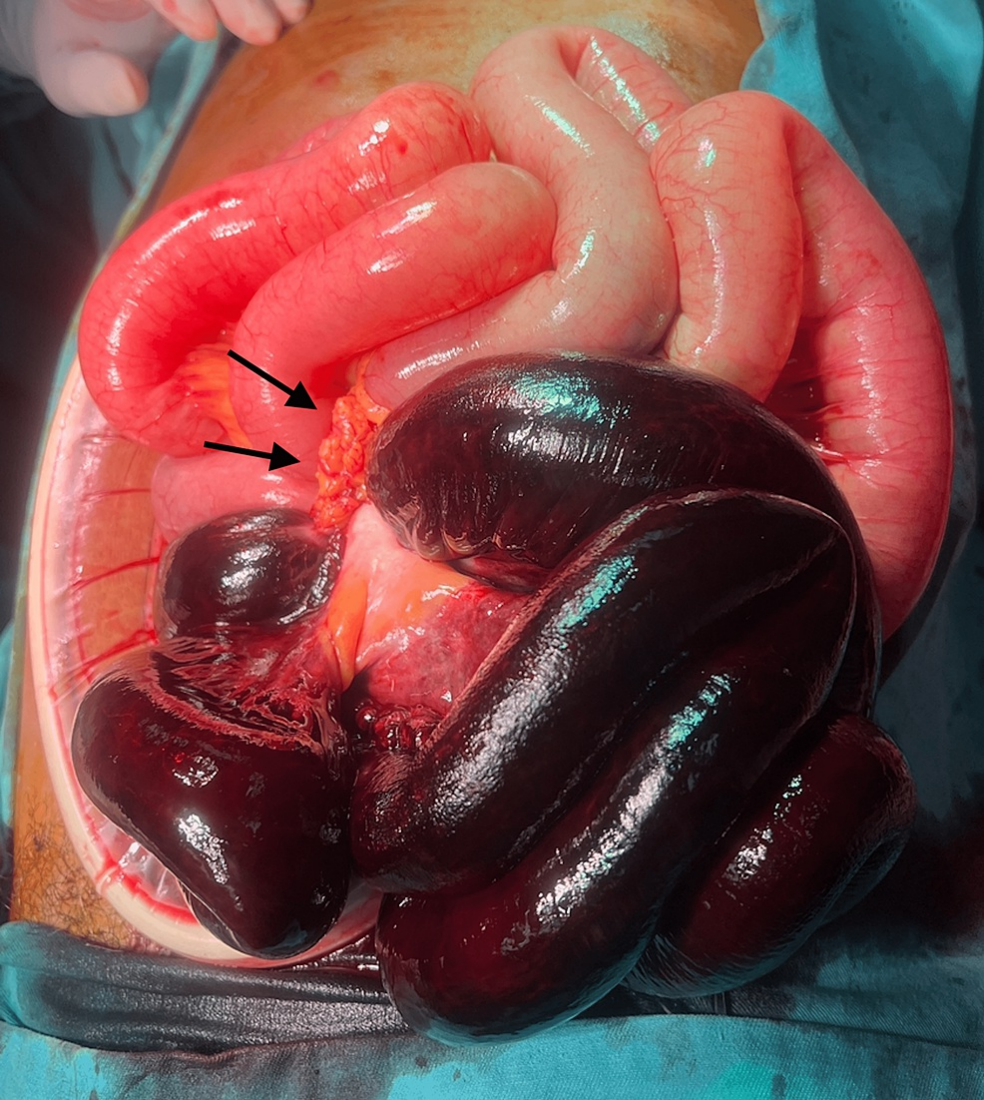
Intraoperative findings showing the omental band as the constriction point (black arrow), with herniation of infarcted ileal loops through it.

**Figure 3:**
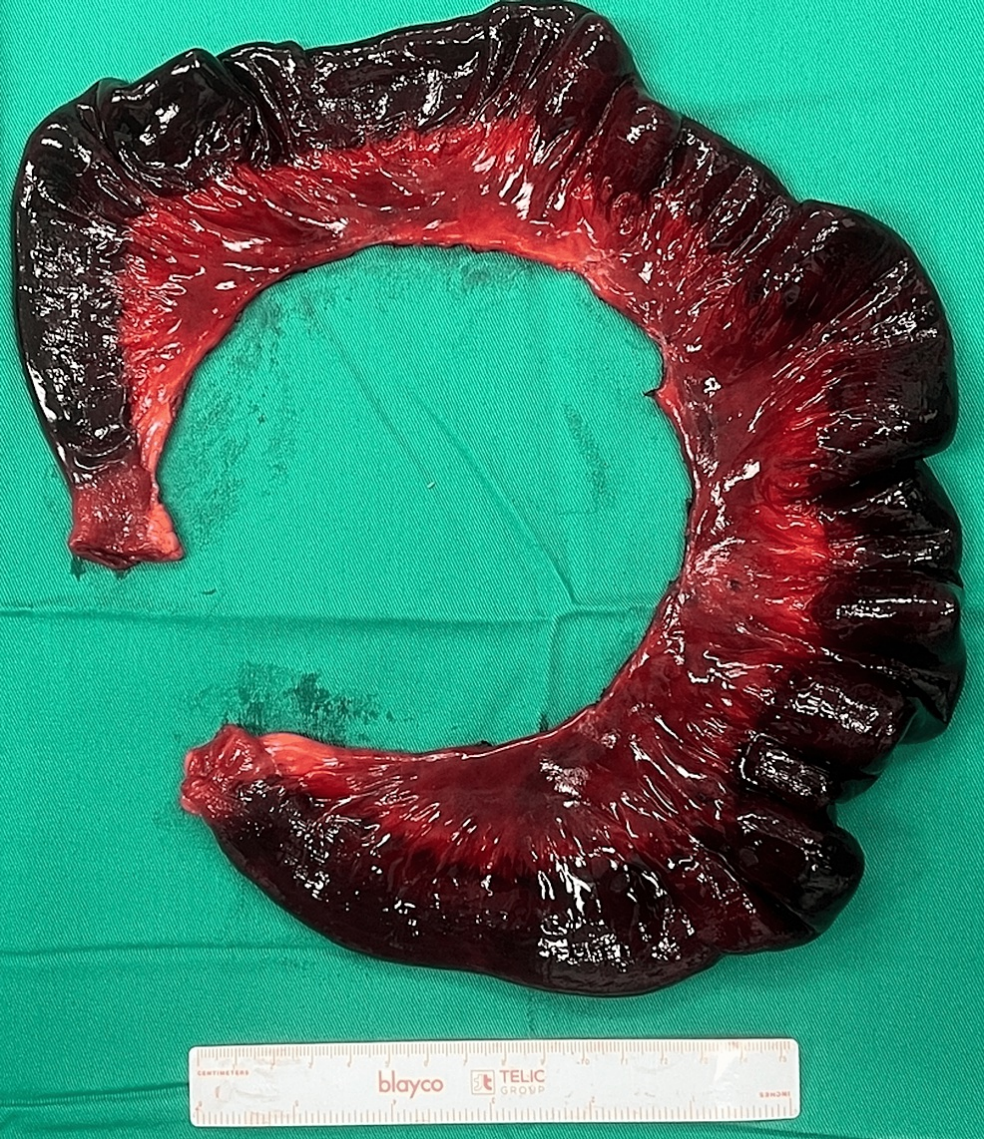
The specimen of resected infarcted ileal loops.

The diagnosis of internal herniation through an omental band leading to ileal ischemia was confirmed during emergency laparotomy as shown in Figure 2. The patient underwent resection of the gangrenous segment (Figure 3) and primary anastomosis. Postoperative recovery was uneventful, and the patient experienced relief from abdominal pain and resolution of bowel obstruction symptoms. Histology of the resected specimen shows infarcted small bowel. While surgical history or trauma is often associated with this condition, our case highlights the importance of considering omental hernia even in the absence of such risk factors. Radiological imaging, particularly CT abdomen, plays a vital role in accurately identifying the location and cause of obstruction. Key CT features, including abnormally clustered bowel loops and a converging appearance of mesenteric fat and vessels, aid in the diagnosis.

## Discussion

Omental band adhesion resulting in acute obstruction of the small bowel within the peritoneal cavity is a recognized condition. The formation of omental bands can be attributed to factors such as congenital factors, previous surgeries, or inflammation [5]. Strangulation caused by adhesional omental bands can lead to obstruction in the form of closed loops, compromising blood supply and resulting in ischemia, infarction, and gangrene of the small bowel. Although adhesional omental bands causing small bowel obstruction are relatively common, their occurrence in elderly patients with no prior abdominal surgeries is unique. The underlying mechanisms for this condition in the absence of previous surgeries are still not clearly understood. Possible factors that may contribute include age-related changes in the omentum, increased susceptibility to adhesions, or hidden factors like subclinical inflammation. 

In our case, the most plausible explanation considering the patient's age and the absence of previous abdominal surgeries or inflammatory conditions is a congenital condition. It is worth noting that omental bands can occasionally lead to the formation of defects, serving as potential spaces for herniation of the intestines, as observed in our case. This narrow space acts as a constriction point, resulting in strangulation. A significant portion of the ileal loops had herniated through this space, highlighting an unusual cause of bowel strangulation, where the constriction is due to an omental band adhesion causing closed loop small bowel obstruction and gangrene.

While there are various causes of small bowel obstruction, the clinical presentation rarely provides a definitive etiology. CT has proven to be valuable in identifying the location, level, and cause of the obstruction. Identifying adhesions as the cause of small bowel obstruction remains a diagnosis made by excluding other causes, relying on the observation of a sudden change in bowel caliber without evidence of alternative obstructive causes [6]. CT features typically associated with internal hernias include abnormal clusters of bowel loops and mesenteric vascular abnormalities such as twisting and whirling [7]. In our case, we observed clustered C-shaped loops of the distal ileum converging toward a mesenteric defect in the right paramedian region. The radiological diagnosis of an internal hernia was established based on the identification of a sac-like mass of dilated small bowel loops within the right hemiabdomen region, taking into account the patient's clinical history of no prior abdominal surgery. CT played a crucial role in enabling the preoperative diagnosis of an internal hernia and guiding early management. Radiologists should remain vigilant about the possibility of a congenital band causing herniation of bowel loops, particularly in elderly patients presenting with small bowel obstruction even in the absence of prior abdominal surgery or trauma.

## Conclusions

In conclusion, an adhesional omental band with small bowel obstruction in an elderly patient with a virgin abdomen presents a unique clinical scenario. The underlying mechanisms of this condition in the absence of prior abdominal surgeries remain uncertain, potentially involving age-related changes in the omentum or other contributing factors. Imaging techniques, particularly CT, play a vital role in diagnosing and managing such cases. Increased awareness and consideration of congenital bands causing herniation of bowel loops are crucial for accurate diagnosis and timely intervention in patients with small bowel obstruction and no previous abdominal surgeries.
